# Immune-Mediated Therapies for Liver Cancer

**DOI:** 10.3390/genes8020076

**Published:** 2017-02-17

**Authors:** Rajagopal N. Aravalli, Clifford J. Steer

**Affiliations:** 1 Department of Electrical and Computer Engineering, University of Minnesota, 200 Union Street S.E., Minneapolis, MN 55455, USA; 2 Departments of Medicine and Genetics, Cell Biology and Development, University of Minnesota, 420 Delaware Street S.E., Minneapolis, MN 55455, USA; steer001@umn.edu

**Keywords:** adaptive immunity, adoptive cell transfer, cancer vaccines, cholangiocarcinoma, hepatocellular carcinoma, immune checkpoint blockade, immunotherapy, innate immunity, tumor immunology

## Abstract

In recent years, immunotherapy has gained renewed interest as an alternative therapeutic approach for solid tumors. Its premise is based on harnessing the power of the host immune system to destroy tumor cells. Development of immune-mediated therapies, such as vaccines, adoptive transfer of autologous immune cells, and stimulation of host immunity by targeting tumor-evasive mechanisms have advanced cancer immunotherapy. In addition, studies on innate immunity and mechanisms of immune evasion have enhanced our understanding on the immunology of liver cancer. Preclinical and clinical studies with immune-mediated therapies have shown potential benefits in patients with liver cancer. In this review, we summarize current knowledge and recent developments in tumor immunology by focusing on two main primary liver cancers: hepatocellular carcinoma and cholangiocarcinoma.

## 1. Introduction

The major primary liver cancers can be classified as hepatocellular carcinoma (HCC), cholangiocarcinoma (CCA), hepatoblastoma, and angiosarcoma. Among these, HCC is the most common form accounting for almost 80% of all primary liver cancers. It is the third leading cause of cancer-related deaths worldwide [1]. HCC commonly develops as a consequence of end-stage liver disease, and is associated with cirrhosis in >90% of cases. A variety of risk factors are known to be causative for HCC. These include infection with hepatitis viruses, aflatoxin B, tobacco, vinyl chloride, heavy alcohol intake, non-alcoholic fatty liver disease (NAFLD), hemochromatosis, and diabetes [2]. In prototypic tumors, when unresolved, low-grade inflammation associated with hepatotropic viruses (HBV, hepatitis B virus; HCV, hepatitis C virus), or intoxications such as alcohol promotes liver cell death. In this pathogenic process, repetitive cycles of cell death and compensatory hepatocellular proliferation cause DNA damage in the presence of mutagenic factors and oncogenic mutations. This toxic environment favors neoplastic transformation and the emergence of HCC. Since it is frequently diagnosed at an advanced stage, HCC has a very poor prognosis rendering current treatment modalities rather ineffective. Tumor resection, chemo- and radiation therapy, percutaneous ethanol injection, radiofrequency ablation (RFA), various embolization procedures, and sorafenib are being used in the clinic to treat patients with liver cancer [2]. However, recurrence is quite common in patients who have had a resection and survival rates range from 30% to 40% at five-year post-surgery.

Cholangiocarcinoma (CCA) is a rare tumor that arises from the neoplastic transformation of cholangiocytes, the epithelial cells that line the intra- and extra-hepatic bile ducts [3]. It is the most common biliary tract cancer and accounts for 10%–20% of all hepatobiliary malignancies [4]. Infection with liver flukes *Clonorchis sinensis* and *Opistorchis viverrini*, hepatitis viruses (HBV and HCV), and primary sclerosing cholangitis (PSC) are major risk factors for CCA [3,5]. Chronic inflammation of biliary ducts, hepatitis, thorotrast are also other known risk factors [6]. Based on their anatomic location within the biliary tree, CCAs are classified as intrahepatic, extrahepatic and perihilar. CCA is difficult to diagnose at an early stage, has poor prognosis, and its incidence is rising rapidly [7]. Current treatment options for CCA include radiotherapy and surgical resection, but the rate of recurrence even after such aggressive therapy is very high. While surgery is used as a curative treatment in the early stages, radiotherapy is more commonly employed to treat CCA. Chemotherapy has not yet been established as a therapeutic option due to very limited effects, and is used only as a last resort treatment option. Administration of gemcitabine either alone or in combination with cisplatin, capecitabine, and oxaliplatin has shown limited efficacy [8]. Overall, liver transplantation remains the best viable treatment option for both HCC and CCA, but is limited due to the severe shortage of available donor livers [9,10].

## 2. Immunobiology of the Liver

**Figure 1:**
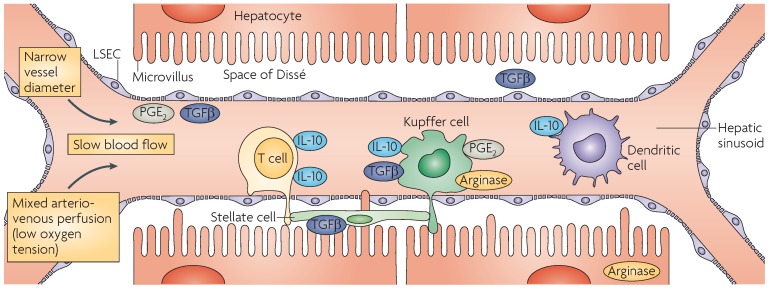
Anatomical location of hepatic antigen-presenting cells (APCs) and the factors that regulate their function. Branches of the hepatic artery merge with sinusoidal vessels carrying blood from the portal vein in the liver, resulting in a mixed arterio-venous perfusion of the liver with low oxygen tension. Owing to extensive branching of portal vessels into liver sinusoids, and the accompanying increase in cumulative vessel diameter, the hepatic microcirculation is characterized by low pressure and slow, sometimes irregular, blood flow. Together with the narrow diameter of hepatic sinusoids, this facilitates the interaction of circulating leukocytes with hepatic sinusoidal cell populations. The hepatic sinusoids are lined by a population of microvascular liver sinusoidal endothelial cells (LSECs) that separate hepatocytes and hepatic stellate cells (HSCs) (all of which function as APCs) from leukocytes circulating through the liver in the blood. Fenestrations in the LSEC lining allow the passive exchange of molecules between the Space of Dissé and the blood, as well as direct contact of lymphocyte filopodia with hepatocyte microvilli. The liver interstitium is highly enriched in cells of the innate immune system (such as antigen-presenting DCs, KCs, NK and NKT cells, and in T cells, which participate in adaptive immune responses. Mediators produced by both parenchymal and non-parenchymal cells, including interleukin-10 (IL-10), transforming growth factor-β (TGFβ), arginase, and prostaglandin E_2_ (PGE_2_), regulate immune function within the liver. Reprinted with permission from NPG [13].

The liver is a lymphoid organ with important metabolic, biosynthetic, digestive, and detoxifying functions [11]. It receives blood from both the systemic circulation via the hepatic artery and the intestine via the portal vein (Figure 1) [11,12,13]. Its major focus is metabolic activity, where the products of digestion are processed and dangerous foreign chemicals are detoxified [11]. Since the liver is exposed continuously to viruses, bacteria, and gut flora, the hepatic environment establishes a balance between immunological tolerance to maintain homeostasis and hepatic immunity against invading pathogens. Hepatocytes and cholangiocytes (bile duct cells) are the main cell types that comprise ~80% of the all liver cells. Non-parenchymal cells in the liver include hepatic stellate cells (HSCs), liver sinusoidal endothelial cells (LSECs), Kupffer cells (KCs), and dendritic cells (DCs). The liver contains a diverse population of both innate and adaptive immune cells [14]. In the normal liver, natural killer (NK) and NK-T (NKT) cells, DCs, LSECs, and KCs present the antigen in the context of inhibitory cell surface ligands and immunosuppressive cytokines, thereby maintaining the liver’s tolerance to various antigens [11,13]. Pathogens such as hepatitis viruses (HBV and HCV) are able to subvert this immunity and establish persistent infection. NK and NKT cells present a first line of immune defense against invading pathogens. Circulating lymphocytes contact the antigen presenting cells (APCs) such as KCs and DCs to modulate immune responses and contribute to the antigen-specific tolerance of the liver [12]. 

### 2.1. Hepatocytes

Immunological functions of hepatocytes have been evaluated in a number of studies. The combination of allogeneic hepatocytes with T cells, resulted in their activation, followed by the apoptosis of T cells in vitro [15]. Similar results were obtained when CD8^+^ T cells from T-cell receptor transgenic mice were exposed to hepatocytes that expressed H2-Kb alloantigen [16]. Analogous studies in vivo using alloantigens also showed that hepatocytes induce T cell activation and apoptosis [17]. When a non-self-antigen was expressed from an adeno-associated viral (AAV) vector in hepatocytes, T-cell activation and cytotoxic effector functions were observed suggesting full functional differentiation of T cells [18]. CD8^+^ T cells can be activated while they are in the intravascular space to perform effector functions against hepatocyte-specific antigens [19], and interact with hepatocytes through fenestrations in LSECs [20].

Hepatocyte expression of major histocompatibility class (MHC) I antigens is generally considered very low, and they do not normally express MHC class II antigens. However, it was recently shown that hepatocytes express MHC class I molecules at levels comparable to that of splenocytes, and efficiently use transporters associated with antigen processing, tapasin (TPN), and low molecular weight polypeptide proteasome subunit components of antigen processing and presentation pathways [21]. They do not express MHC class I loading complex factors LMP7, LMP10, PA28-α, and PA38-β, while TAP1 and TAP2 are expressed in low abundance. In chronic liver diseases, however, both class I and class II antigens can be detected on hepatocytes. Similarly, hepatocytes express MHC class I complexes when exposed to IFN-γ and *Listeria monocytogenes* with high levels of stable class I trimers displayed on their cell surface [21]. Recently it was shown that the expression of MHC class I-related chain A (MICA) was highly elevated after 27 days of infection in human hepatocytes, suggesting that they can act as APCs [22]. Overexpression of class II transactivator molecule, a key transcriptional regulator of MHC class II gene expression, in hepatocytes resulted in the surface expression of MHC class II molecules and activation of CD4^+^ T cells [23]. In this case, MHC II-expressing hepatocytes were able to process antigens and stimulate Th1 or Th2 cell lines, and there was no sign of hepatic inflammation and autoimmune liver disease in transgenic mice expressing class II molecules. Collectively, these findings demonstrated that hepatocytes can also act as APCs.

### 2.2. Cholangiocytes

Even though cholangiocytes express many molecules that are often linked to APC function, there is no evidence to support the prospect that they activate T cells [24]. However, bile duct cells were shown to express MHC I and II, CD40, CD80, and CD86 molecules [25], and secrete CXCL16 that promote T-cell adhesion to epithelial cells [26].

### 2.3. Kupffer Cells

KCs are the resident macrophages of the liver. They are a unique cell type that are typically radio-resistant and difficult to isolate from tissue, even after collagenase digestion [24]. KCs induce immunotolerance under physiological conditions. For instance, they secrete immunosuppressive prostaglandin E2 (PGE2) under metabolic stress [27], and interleukin-10 (IL-10) when stimulated with lipopolysaccharide (LPS) [28]. They also express MHC classes I and II, and co-stimulatory molecules at low density [29]. Continuous exposure to endotoxin (LPS) limits the ability of KCs to activate T cells [29], and PGE2 released by KCs abrogates CD4^+^ T-cell activation [30]. In response to reactive oxygen species, however, KCs produce MHC class II molecules and act as APCs [31]. Thus, KCs are able to switch their immunological role from tolerance-inducing APCs to immunogenic APCs, and from inactivators to activators of NK cells when exposed to certain bacteria, such as *Borrelia burgdorferi* [24,32].

### 2.4. Dendritic Cells

The liver contains multiple populations of DCs, including plasmactyoid (pDC), myeloid (mDC), and lymphoid-derived (CD8α^+^) DCs. Both pDCs and mDCs are weak APCs because they are immature cells, whereas CD8α^+^ DCs are powerful APCs [24]. mDCs are characterized by their expression of CD11b and CD11c and lack CD8α and B220 expression. On the other hand, mouse liver pDCs are B220^+^ and express CD11c at lower levels than mDCs, whereas human pDCs lack CD11c but express blood DC antigen-2 (BDCA-2) [24]. A comparison of liver mDCs with skin-derived mDCs showed that liver cells secrete greater amounts of interleukins IL-10 and IL-4, whereas skin DCs were potent stimulators of interferon-γ (IFN-γ) and IL-4 [33]. Moreover, liver mDCs were found to be less effective at stimulating T-cell proliferation suggesting that hepatic mDCs predispose T cells towards tolerance [24]. Even though pDCs are not effective at stimulating T-cell activation [34], growth factors and Toll-like receptor (TLR) signaling can induce maturation of these cells into APCs and stimulate T cells [35].

### 2.5. Liver Sinusoidal Endothelial Cells

LSECs, which account for almost half of the non-parenchymal cells, induce immune tolerance via their expression of MHC I and II, as well as costimulatory molecules CD40, CD80, and CD86 [36]. They are able to eliminate viruses, colloids, and macromolecular waste from circulation through the expression of acetylated low density lipoprotein and mannosylated protein receptors [37]. Antigen presentation to T cells by LSECs via MHCs results in the up-regulation of specific molecules like the programmed death ligand 1 (PDL-1) which binds to its cognate receptor PD-1 causing T-cell tolerance [24]. However, the exposure to endotoxin reduces the ability of LSECs to activate antigen-specific CD4^+^ T cells [38]. In addition, IL-10 secreted by KCs can enhance the antigen presentation capacity of LSECs [30].

### 2.6. Hepatic Stellate Cells

HSCs, also termed Ito cells, reside in the Space of Dissé and regulate blood flow through the sinusoids [39]. In the murine liver, HSCs express CD1d, and low levels of CD11c and MHC class II molecules but not MHC class I [40]. However, a tiny population of CD11c-high cells do exist and they activate NKT cells and T cells in the presence of α-glactosyl ceramide [40]. Additional data from this study showed that HSCs presented antigenic peptides to CD8^+^ and CD4^+^ T cells and mediated cross-priming of CD8^+^ cells. Further, rat HSCs were reported to stimulate antigen-specific CD8^+^ responses [41], whereas human HSCs induced very little proliferation of CD8^+^ T cells [42]. Additional studies are needed to ascertain the function of HSCs as APCs.

## 3. Tumor Microenvironment

Carcinogenesis is a multistage process that appears to involve the transformation of a normal differentiated or stem-like cell into a preneoplastic lesion that develops into a malignant tumor [14]. Most of the gene mutations are somatic and occur as sporadic events; and some cancers could arise from epigenetic changes, such as DNA methylation and histone modification [43]. The tumor microenvironment is a complex interplay of T cells, B cells, macrophages, and hepatic cells resulting in the activation of multiple cellular signaling cascades [2].

### 3.1. Tumor-Infiltrating Lymphocytes

There is growing evidence to suggest that the gradual accrual of genetic changes and mutations in preneoplastic hepatocytes causes a cellular transformation resulting in the development of HCC [44]. Interactions of immune cells in the tumor microenvironment also play a critical role in cancer development, and the presence of intratumoral T and B cells, termed tumor infiltrating lymphocytes (TILs), correlates with improved clinical outcomes [45,46,47,48,49]. TILs in HCC include subpopulations of CD3^+^, CD4^+^, and CD8^+^ T cells [48]. The presence of CD8^+^ T cells in tumors is associated with better outcomes, whereas the presence of regulatory T cells (Tregs) is associated with poor prognosis [50]. Although antitumor effects of T cells seem to be impaired in HCC, the underlying mechanism(s) for this phenomenon is unclear [48]. Both in vitro and in vivo studies have shown that Tregs are involved in the deterioration of antitumor effects of T-lymphocytes. In HCC, TILs interact with a number of tumor-associated antigens (TAAs), such as glypican-3 (GPC3), α-fetoprotein (AFP), telomerase reverse transcriptase (TERT), synovial sarcoma X breakpoint 2 (SSX-2), melanoma antigen gene-A (MAGE-A), and New York esophageal squamous cell carcinoma 1 (NY-ESO-1) [51].

### 3.2. Tumor-Associated Macrophages

In the tumor microenvironment, tumor-associated macrophages (TAMs) are “polarized” into M2 mononuclear phagocyte-like cells by various cytokines (IL-4, IL-10, and transforming growth factor β (TGF-β)) [52]. These M2-like TAMs, in turn, express cytokines (IL-10 and TGF-β), chemokines (CCL17, CCL22, and CCL24), vascular endothelial growth factor (VEGF), and epidermal growth factor (EGF) to recruit Tregs and promote angiogenesis [53,54]. KCs are liver-specific TAMs, and are able to impair CD8^+^ cytotoxic T lymphocyte (CTL)-mediated immune responses through PD-L1, which interacts with programmed death 1 (PD-1), a cell surface protein of CD8^+^ T cells [55]. Moreover, when stimulated with pro-inflammatory cytokines (IL-1β, TNF-α, and PDGF), KCs and HSCs produce osteopontin, which plays a pivotal role in various cell signaling pathways, promotes inflammation, tumor progression, and metastasis [56,57]. Similar transitions in bile duct cells were shown to also result in the development of CCA [58].

### 3.3. Innate Immunity in the Liver

The innate immune system provides the first line of defense to limit infection in the liver by recognizing conserved structural motifs, termed pathogen-associated molecular patterns (PAMPs), on the surface of various pathogens. TLRs are key players in this process [34] and also recognize damage-associated molecular patterns (DAMPs) on dying host cells [59]. Although hepatocytes express all TLRs at low levels, they only respond to TLR2 and TLR4 ligands [60]. Under inflammatory conditions, however, hepatocyte response to TLR2 ligands is significantly enhanced but interestingly not so against TLR4 ligands [61]. LSECs express mRNAs of TLRs 1–9, and respond to various TLR ligands by producing TNF-α, IL-6, and IFN-β [62,63]. KCs express all TLRs and respond to a variety of TLR ligands by producing TNF-α and IL-6 [64,65]. KCs activated in response to TLR2 and TLR4 produced the immunosuppressive IL-10, and in turn, were able to suppress IL-18-dependent NK cell activation [60]. When stimulated with ligands for TLRs 1, 2, 4, and 6, KCs also produced IFN-γ and promoted the proliferation of T cells [62]. In response to TLR3 and TLR4 ligands, they secreted IFN-β, and with ligands against TLR1 and TLR8, displayed a high level of MHC class II expression. HSCs express low levels of TLR4 and TLR9, but activation of TLR4 has been shown to induce the expression of TLR2 [62]. In human HSCs, TLR4 activation resulted in the production of CCL2, CCL3, and CCL4 [66], and their expression of TGF-β was implicated in the promotion of hepatic fibrosis [67]. Activation of TLR9 by DAMPs induced the differentiation of HSCs and increased the production of collagen [68]. The potential of innate immune mechanisms must and can be harnessed to target infections, such as HBV and HCV that cause HCC [69].

## 4. Immunotherapeutic Approaches to Treat Liver Cancer

In the past decade, advances in our understanding of the immunobiology of the liver has led to the development of immunotherapeutic strategies, including vaccination, antibody-based treatments, adoptive cell therapy, immune checkpoint blockade, and cytokine targeting. Some of these have produced positive results and paved the way for their use in the clinical setting (Table 1).

### 4.1. Vaccines

Several vaccines targeting TAAs that were identified in HCC are being used for cancer therapy [51]. Among them, GPC-3, a member of the glypican family of heparin sulfate proteoglycans, was found to be overexpressed in HCC patients and was associated with poor prognosis [70]. In a phase I clinical trial, when GPC3_298-306_ peptide was used as a TAA in HLA-A*24-positive patients and GPC3_144-152_ peptide in HLA-A*02-positive patients, only one out of 33 patients manifested a partial response and all patients had marked intratumoral infiltration of CD8^+^ T cells [71]. In a phase II clinical trial with GPC-3 vaccine, recurrence rates were lower in 35 out of 64 patients that had undergone surgery or RFA prior to receiving the vaccine, and the GPC3 peptide vaccine improved one-year recurrence rate [72]. On the other hand, a similar phase II trial using hTERT peptide did not demonstrate any CTL activity [73].

Recently the development of an in situ cancer vaccine InCVAX for the treatment of HCC has been reported [74,75]. InCVAX works by stimulating robust antitumor immune reactions via a two-pronged approach. This includes a thermal treatment of tumors, such as with a laser, followed by administration of *N*-dihydro-galacto-chitosan (NDGC). Thermal treatment releases antigens and increases immunogenicity, while NDGC acts as a potent immune activator [74]. In that study, the strategy of local immunological cell death with DC activation with NDGC was found to be effective in a murine HCC model. Tumor infiltration of CD3^+^, CD4^+^, and CD8^+^ cells was observed after the injection of InCVAX, which indicated the in situ elimination of the tumor [74]. A vaccine containing the epitope of α-fetoprotein (AFP) linked with a heat shock protein 70 epitope also elicited anti-tumoral immunity by activating AFP-specific CD8^+^ T cells and was effective in reducing tumors in mice [76]. These vaccines need further improvement in determining therapeutic efficacy on tumor regression and recurrence.

In contrast to HCC, a limited number of protocols have been conducted to treat CCA. In one study, 36 patients with intrahepatic CCA were vaccinated with autologous tumor lysate pulsed DCs, together with the transfer of activated T cells [77]. A five-year progression-free survival and overall survival in these patients were substantially higher than in 26 patients who received curative resection alone (progression-free survival: 18.3 months vs. 7.7 months and overall survival: 31.9 months vs. 17.4 months). These findings suggested that a combination of DC vaccine and T-cell transfer might prevent recurrence and achieve long(er)-term survival in CCA patients [77].

Another potential candidate for immunization is the mucin protein 1 (MUC1), a glycoprotein that is overexpressed in 59%–77% of CCA, and its expression correlated with the patients′ overall survival rate [78,79,80]. In a non-randomized trial, DC vaccine targeting MUC1 showed a modest increase in the median survival time and had no adverse effects on patients, suggesting that it was safe for administration in humans [81]. However, further studies are needed to evaluate its effect, in particular in the early disease stage and in the absence of immunosuppressive therapy.

### 4.2. Adoptive Cell Therapy

**Figure 2:**
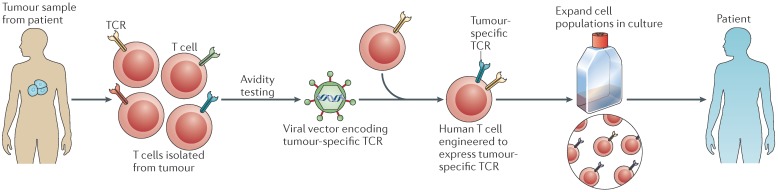
Adoptive cell transfer. T cells can be genetically engineered to recognize tumor-associated antigens in various ways. If a patient expresses a tumor-associated antigen (TAA) that is recognized by an available receptor structure, autologous T cells can be genetically engineered to express the desired receptor. New receptors can be generated in a variety of ways. T cells can be identified and cloned from patients with particularly good antitumor responses. Their T cell receptors (TCRs) can be cloned and inserted into retroviruses or lentiviruses, which are then used to infect autologous T cells from the patient to be treated. Reproduced with permission from NPG with modifications [99].

Adoptive cell therapy (ACT) involves engineering patients′ own immune cells to recognize and destroy their tumors. In this approach, T cells from a healthy patient′s blood are collected and are genetically modified to express an artificial receptor consisting of the variable fragment of an antibody specific for a cell surface molecule involved in T cell signaling. Such engineered receptors (termed chimeric antigen receptors (CARs)) allow T cells to recognize specific antigens on tumor cells (Figure 2). CAR-T cells are then propagated in cell culture until they reach several billions in number, and injected back into the patient′s blood stream. After infusion, CAR-T cells are designed to recognize tumor cells and kill them. CAR-T cells have advantages over T-cell receptor-modified T (TCR-T) cells in that they recognize tumor cells without MHC restrictions [82], which will allow improved patient targeting and overcoming tumor escape mechanisms of MHC loss. This ACT approach has shown promise in recent clinical trials of patients with acute lymphoblastic leukemia [83], acute myeloid leukemia [84,85], and gastrointestinal cancer [86]. A clear distinction between autologous transfer using endogenous T cells and an allogeneic approach should be noted. The former approach involves modified T cell receptors that recognize peptides with HLA restrictions, whereas in the latter there is no HLA restriction and it involves the transfer of cells from an immunized individual to a non-immune recipient. This difference is critical for assessing the extent of toxicities associated with off-tumor and on-target effects.

To date, very few studies have been conducted on the application of CAR-T cells to HCC and CCA immunotherapy. In one study, T cells expressing CAR targeted to GPC-3 have successfully eliminated GPC3-positive cells in vitro and in orthotopic Huh-7 xenografts in mice [87]. However, substantial risks and toxicities are associated with the use of CAR-T cells, as they release massive amounts of cytokines into the patient’s blood stream. These include, but are not limited, to cytokine release syndrome, B-cell aplasia, and tumor lysis syndrome [85]. Clinical studies of solid tumors using CAR-T cells have also shown considerable toxicity towards normal tissues [88,89]. In this regard, studying the efficacy and safety of CAR-T cells in animal models is necessary for the validation of CAR constructs under therapeutic settings in the treatment of solid tumors [82]. Distinguishing therapeutic efficacy from off-tumor toxicity would be of the utmost importance for ACT, as are further modifications of CAR-T cells, identifying better targets, and improving preconditioning regimens.

Cytokine-induced cells, that include activated T cells and NK cells, have shown clinical benefits in adoptive immunotherapy [90,91,92]. Randomized clinical trials of adjuvant immunotherapy using cytokine-induced killer (CIK) cells showed mixed results for HCC. In one study, no improvement in patient survival was observed even though there was an increase in the recurrence-free survival [93]. Others showed that lymphocyte infusions (CD3^+^, CD3^+^/HLA-DR^+^, CD4^+^, and CD8^+^) lowered recurrence and improved recurrence-free outcomes after surgery in HCC patients [94]; CIK therapy improved overall survival in HCC patients [95]; and infusion of CD3^+^/CD56^+^ and CD3^+^/CD56^−^ T cells, as well as CD3^−^/CD56^+^ NKT cells increased recurrence-free and overall survival in HCC patients who underwent surgical resection or RFA or percutaneous ethanol injection [96]. Two other studies showed that CIK therapy also reduced 1-year recurrence rates in patients who received a combination treatment with RFA and TACE [97], and in those HCC patients unsuitable for surgery [98]. Collectively, these clinical trials suggested that CIK therapy might be more suitable for patients who have early stage HCC that those with advanced disease.

There have been a limited number of studies conducted with CIK cells for immunotherapy. In one study, human CIK cells comprising of CD3^+^ T cells and CD3^+^/CD56^+^ T cells were able to reduce the growth of inoculated CCA cells in SCID mice [100]. In another study, CIK cells expressing inducible co-stimulator had profound cytotoxic effects on CCA cells in vitro and in vivo [101]. CIK cells in combination with cetuximab, an antibody that targets epidermal growth factor receptor, significantly enhanced cytotoxicity of human CCA cells in vitro than when used alone [102]. Further studies are needed to evaluate the clinical use of CIK cells for the treatment of CCA.

Another interesting line of research is the combination of peptide vaccine together with ACT. A case report using a personalized peptide-vaccine that elicited strong immune response was recently shown to be effective in a patient with metastatic CCA [103]. Immunotherapy using peptide vaccines together with adoptive transfer of T cells is also emerging as an exciting option [86,104].

### 4.3. Immune Checkpoint Blockade

**Figure 3:**
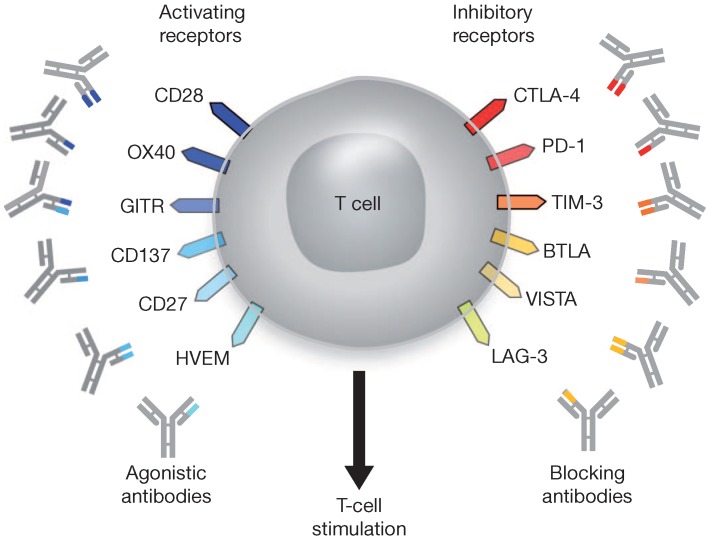
T cell targets for immunoregulatory therapy using antibodies. In addition to specific antigen recognition through the TCR, T-cell activation is regulated through a balance of positive and negative signals provided by co-stimulatory receptors. These surface proteins are typically members of either the TNF receptor or B7 superfamilies. Agonistic antibodies directed against activating co-stimulatory molecules and blocking antibodies against negative co-stimulatory molecules might enhance T-cell stimulation to promote tumor destruction. Reprinted with permission from NPG [109].

In recent years, a blockade of checkpoint molecules that block immune responses against tumor cells, such as PD-1, PD-L1 and CTLA-4, has emerged as a novel therapeutic approach in oncology [105]. Antibodies that target these molecules have been developed, and they have shown significant efficacy (Figure 3). Among these, pembrolizumab and nivolumab target PD-1, and tremelimumab and ipilimumab target CTLA-4 [106]. Pembrolizumab has been approved by the Food and Drug Administration (FDA) in the United States for the treatment of metastatic melanoma (MM) and non-small cell lung cancer (NSCLC); nivolumab for MM, NSCLC and renal cell carcinoma, and ipilimumab for MM, either as single agents or in combination [107]. These were also approved by the European Medicines Agency [108].

#### 4.3.1. Programmed Death-1

PD-1, a negative co-stimulatory molecule of the CD28 immunoglobulin superfamily of transmembrane receptors, is a strong inhibitor of T cell response. Therefore, blocking its function is a strategy for immunotherapy. PD-L1 and PD-L2, members of the B7 costimulatory molecule family, are ligands for PD-1. Engagement of PD-L1 and PD-L2 with PD-1 inhibits T cell receptor-mediated lymphocyte proliferation and cytokine production by CD4^+^ T cells [110,111]. A clinical trial using tremelimumab in 17 HCC patients with liver cirrhosis and HCV showed that three patients developed a partial response with a median survival of 8.2 months, and HCV-specific T cell responses did not correlate with tumor regression [112]. Therefore, TAA-specific CD8^+^ T cell responses could reduce the recurrence of HCC suggesting that immunotherapy to induce TAA-specific CTLs by such means as peptide vaccines might be an effective clinical application in HCC patients after local therapy [113]. In another randomized trial, a single dose of the antibody nivolumab (BMS-936558) was administered to patients with chronic HCV infection. There were no significant side effects, and one-third of the recipients had reduced viral loads [114]. Blocking PD-1 function might also improve disease outcomes in CCA as shown recently [115]. Patients with high levels of soluble PD-L1 had worse overall survival than those with low levels of the ligand. In another study, PD-L1 and MHC class I expression were elevated in eight and 11 out of 27 intrahepatic CCA tumors samples, respectively; whereas all tumor samples had infiltration of CD8^+^ T cells [116]. Results from this study also showed that the defects in MHC class I antigen expression and high PD-L1 expression by tumor cells could potentially provide them with an immune escape mechanism. Therefore, immunotherapy with antibodies against PD-1 in patients with normal MHC class I expression might be an effective strategy to treat intrahepatic CCA [116].

#### 4.3.2. CD160

CD160, a negative co-stimulatory molecule, was found to be associated with T cell exhaustion in patients with chronic HCV infection where it was overexpressed on HCV-specific CD8^+^ T cells in the peripheral blood [117]. However, it is not overexpressed in intrahepatic HCV-specific CD8^+^ T cells [118]. Additional studies are needed to understand the importance of CD160/CD160L blockade.

#### 4.3.3. Natural Killer Cell Receptor 2B4

NK cell receptor 2B4 was also found to be overexpressed in the blood of patients with chronic HCV [117]. Its ligand CD48 has a 6 to 8-fold greater affinity to 2B4 than CD2, a molecule implicated in the regulation of NK and T cell activation [119]. Expression of 2B4 on HCV- specific CD8^+^ T cells also correlated with the overexpression of PD-1 [117], which was higher in hepatic cells than in blood cells [118]. Collectively, these findings indicated that the up-regulation of 2B4 is potentially linked to PD-1 expression.

#### 4.3.4. Lymphocyte Activation Gene-3

LAG-3 was another receptor overexpressed on HCV-specific CD8^+^ cells, where it negatively regulated the function of these cells in chronic HCV patients [120]. Blockade of LAG-3 restored T cell effector functions in this cohort. Similar results were obtained in patients with HBV-related HCC [121].

#### 4.3.5. T-Cell Immunoglobulin and Mucin-Domain Containing-3

Tim-3 was identified as a negative regulator of T-helper type 1 immunity through the binding of its ligand galectin-9 [122]. It is up-regulated in CD8^+^ cells of patients with chronic HCV, and its blockade restored the effector function of these cells [123]. Thus, Tim-3 is a prime candidate for immunotherapy.

#### 4.3.6. Glycoprotein-2

In patients with PSC, antibodies against GP-2 were found to be elevated, and was associated with poor patient survival [124]. High levels of anti-GP2 IgA also correlated with the development of CCA in patients with PSC in this study. Therefore, anti-GP2 IgA might be a valuable tool for risk stratification in patients with PSC.

### 4.4. Antiplatelet Therapy

Platelets (also known as thrombocytes) are small enucleated cells that are present in the bloodstream. When vascular injury and damage occurs, platelets are activated resulting the secretion of growth factors such as hepatocyte growth factor (HGF), fibroblast growth factor (FGF), insulin-like growth factor (IGF)-1, VEGF, EGF, and platelet-derived growth factor (PDGF), during a process called thrombosis [125]. Platelets improve liver fibrosis by inactivating HSCs to decrease collagen production and accelerate liver regeneration [126]. Thrombocytosis occurs when platelet numbers are elevated in the blood, and a strong association has been found between HCC and thrombocytosis. In a retrospective study of 1154 HCC patients, it was found that patients with thrombocytosis had higher AFP levels, large tumor volume and high platelet count with short survival rates [127]. A recent study has shown that high platelet count could be a reliable marker for extrahepatic metastasis in early stage HCC following curative treatment [128]. High platelet levels were also reported more recently in CCA [129]. On the other hand, thrombocytopenia, a condition with low platelet count, was also shown to be associated with HCC in a cirrhotic background [130], and correlated with a reduced overall survival in patients with HCC [131]. Collectively, these results demonstrated that platelets play an important role in predicting the overall survival of patients with liver cancer.

Within the liver sinusoids, circulating CD8^+^ T cells induce the arrest of platelet aggregates that are bound to sinusoidal hyaluronan via CD44 [19]. In the mouse model of HBV, CD8^+^ T cells with effector functions control hepatotropic pathogens by crawling along liver sinusoids. Activated platelets mediate CTL-induced liver damage in mouse model of acute viral hepatitis [132]. Administration of aspirin and clopidogrel (Asp/Clo) that block platelet activation prevents HCC and improves survival in a mouse model of chronic HBV infection [133]. In this study, the authors also observed a significant reduction in the progression of liver fibrosis. Similarly, HBV-associated HCC patients who received aspirin and clopidogrel had a better recurrence-free survival and OS than those without the antiplatelet therapy [134]. The mechanism by which platelets interact with T cells is unknown, and antiplatelet therapy could diminish the release of TGF-β as platelets are the main repository for this profibrogenic cytokine [135]. Due to the protective role of platelets against various other infections, such as malaria [136], blocking platelet activation could be risky as antiplatelet therapy could increase the bleeding risk in patients with impaired liver function [137]. Further studies are needed to assess the impact of antiplatelet drugs on liver inflammation and HCC.

## 5. Future Directions

The incidence of liver cancer is rapidly increasing, and the annual health care costs of HCC treatment are skyrocketing [138]. In the United States, the overall median cost to care for a patient with HCV-associated HCC was recently estimated to be almost $180,000/year [139]. Therefore, there is a substantial burden of medical expense of illness associated with liver cancer, and a greater need exists for the development of novel therapies. A number of clinical trials are currently underway for both HCC [140] and CCA [141], which could produce very effective immunotherapies.

A critical avenue to pursue further is the identification of new targets, which requires extensive validation across species, although caution must be exercised in their discovery. For instance, mesothelin was reported as a potential immunotherapeutic target in various cancers. When HCC and CCA specimens were investigated for the expression of mesothelin, none was found in HCC specimens, but one-third of CCA tissues overexpressed mesothelin, as did three distinct CCA cell lines [142]. Addition of sulfatase-1 that targets mesothelin showed very high and specific growth inhibition of these cell lines, suggesting that it could be a potential therapeutic agent for CCA [142]. Even though this finding suggested that sulfatase-1 could act as a tumor suppressor in HCC, caution should be exercised in interpreting it as no mesothelin expression was detected in human HCC specimen.

TGN1412 was developed in the late 1990s as an antagonistic monoclonal antibody against CD28, a key co-stimulator for T-cell responses [143]. Experimental ground work conducted in rats and mice using this antibody has revealed significant promise to treat autoimmune and inflammatory diseases. However, when a single dose of TGN1412 was administered to six healthy volunteers, they all experienced severe cytokine release syndrome with multi-organ failure [144]. The study results demonstrated the potential danger involved in extrapolation of results from animal studies to humans in preclinical studies. Similarly, extrapolation of data from one species to another may not be feasible in case of CAR-T cells, particularly in HLA-restricted peptides, due to the differences between species.

Recent advances in immunotherapy have paved the way towards the goal of not only treating tumor recurrence, but also preventing the development of cancer in cirrhotic livers. Immune tolerance of the liver is still a major problem. However, efforts are being made to circumvent this issue by using immune checkpoint inhibitors. Preclinical studies and clinical trials have demonstrated promising results in this direction. At present, it appears that a combination therapy of vaccines and/or immune checkpoint inhibitors with local ablative therapies is an attractive approach in treating liver cancer. However, safety, toxicity, and efficacy in clinical trials must be addressed before bringing these immunotherapeutic interventions to the bedside.

## Figures and Tables

**Table 1:** Some of the immunotherapeutic strategies currently used in clinical trials to treat liver cancer.

Immunotherapeutic Strategy	Mode of Application	Mechanism of Action
Vaccines	Antigenic peptides/proteins (GPC-3, AFP, NDGC, MUC1) DC-based vaccines, APCs from tumor lysates, InCVAX	Targeting TAAs to overcome immune tolerance by expressing antigenic proteins/peptides or co-stimulatory molecules in DCs or tumor cells
Adoptive cell therapy	CIK infusion, CTL transfer TCR-T cells	Transfer of tumor-specific T cells from a healthy individual into the bloodstream of the patient to be treated after propagating them ex vivo to enhance immune responses
	CAR-T cells, TCR-T cells	Patient-derived T cells are modified to express artificial receptors, propagated in vitro and administered back into the same patient
Immune checkpoint blockade	Antibody (Pembrolizumab Ipilimumab, Nivolumab Tremelimumab, AMP-224 Lambrolizumab, CT-011, and others)	Targeting specific cellular receptors and their ligands (PD-1, PD-L1, CTLA-1, CD160, 2B4, LAG-3, Tim-3, GP-2), and to enhance antigen presentation

## Abbreviations

The following abbreviations are used in this manuscript:

AFP	α-fetoprotein
APC	Antigen-presenting cell
BDCA-2	Blood dendritic cell antigen-2
CAR	Chimeric antigen receptor
CCA	Cholangiocarcinoma
CIK	Cytokine-induced killer cell
CTL	Cytotoxic T-lymphocyte
DC	Dendritic cell
GPC3	Glypican-3
GP-2	Glycoprotein-2
HBV	Hepatitis B virus
HCC	Hepatocellular carcinoma
HCV	Hepatitis C virus
HSC	Hepatic stellate cell
IFN-γ	Interferon-γ
IL-10	Interleukin-10
KC	Kupffer cell
LAG-3	Lymphocyte activation gene-3
LPS	Lipopolysaccharide
LSEC	Liver sinusoidal endothelial cell
MAGE-A	Melanoma antigen gene-A
MHC	Major histocompatibility complex
NAFLD	Non-alcoholic fatty liver disease
NK	Natural killer
NY-ESO-1	New York esophageal squamous cell carcinoma 1
NKT	NK T cell
PD-1	Programmed death 1
PD-L1	Programmed death ligand 1
PGE2	Prostaglandin E2
PSC	Primary sclerosing cholangitis
RFA	Radiofrequency ablation
SSX-2	Synovial sarcoma X breakpoint 2
TAA	Tumor-associated antigen
TAM	Tumor-associated macrophages
TAP-1	Tapasin-1
TCR	T-cell receptor
TERT	Telomerase reverse transcriptase
TGF-β	Transforming growth factor-β
TIL	Tumor-infiltrating lymphocyte
Tim-3	T-cell immunoglobulin and mucin-domain containing-3
TLR	Toll-like receptor
Treg	Regulatory T-cell
